# Prostate tuberculosis mimicking malignancy on ^18^F-FDG PET/CT in a patient with diffuse large B-cell lymphoma: A case report

**DOI:** 10.1097/MD.0000000000038296

**Published:** 2024-05-31

**Authors:** Min Bai, Qinchuan Yu, Ling Yuan, Yuanyuan Zhao, Meijing Zheng, Liping Su, Lieyang Wang

**Affiliations:** a Department of Hematology, Shanxi Province Cancer Hospital/Shanxi Hospital Affiliated to Cancer Hospital, Chinese Academy of Medical Sciences/Cancer Hospital Affiliated to Shanxi Medical University, Taiyuan, Shanxi, China; b Department of PET/CT, Shanxi Province Cancer Hospital/Shanxi Hospital Affiliated to Cancer Hospital, Chinese Academy of Medical Sciences/Cancer Hospital Affiliated to Shanxi Medical University, Taiyuan, Shanxi, China; c Department of Patholology, Shanxi Province Cancer Hospital/Shanxi Hospital Affiliated to Cancer Hospital, Chinese Academy of Medical Sciences/Cancer Hospital Affiliated to Shanxi Medical University, Taiyuan, Shanxi, China.

**Keywords:** ^18^F-FDG, case report, lymphoma, PET/CT, tuberculosis

## Abstract

**Background:**

Prostate tuberculosis (TB) is a rare and often underdiagnosed condition due to its nonspecific symptoms and imaging features, which can mimic malignancies on ^18^F-fluorodeoxyglucose positron emission tomography (PET) scans. This resemblance poses a challenge in differentiating TB from prostate cancer, especially in patients with preexisting tumors such as diffuse large B-cell lymphoma. The purpose of this study is to highlight the importance of considering TB in the differential diagnosis of patients with atypical imaging findings, even in the presence of known malignancies.

**Case:**

We present a case of a 60-year-old man with diffuse large B-cell lymphoma who was initially misdiagnosed with a prostate tumor based on ^18^F-fluorodeoxyglucose PET/computed tomography scans. The subsequent ultrasound-guided prostate biopsy confirmed the presence of prostate TB, not malignancy.

**Conclusions:**

This case report underscores the critical role of considering TB as a potential diagnosis in patients with hematological tumors and atypical imaging results. It serves as a reminder for clinicians to exercise caution when interpreting PET/computed tomography scans and to incorporate TB into their differential diagnoses, thereby avoiding misdiagnosis and inappropriate treatment.

## 1. Introduction

Diffuse large B-cell lymphoma (DLBCL) is the most common subtype of non-Hodgkin lymphoma. It is a highly aggressive malignancy that can arise from any part of the lymphoid system, including the lymph nodes, spleen, bone marrow, and gastrointestinal tract. Prostatic involvement in DLBCL is rare, with an estimated incidence of <1%. We present a case of a 60-year-old man with DLBCL who developed prostatic tuberculosis (TB) during chemotherapy. This case highlights the importance of considering TB as a differential diagnosis in patients with DLBCL, even in the absence of typical clinical symptoms.

## 2. Case report

**Figure 1.**
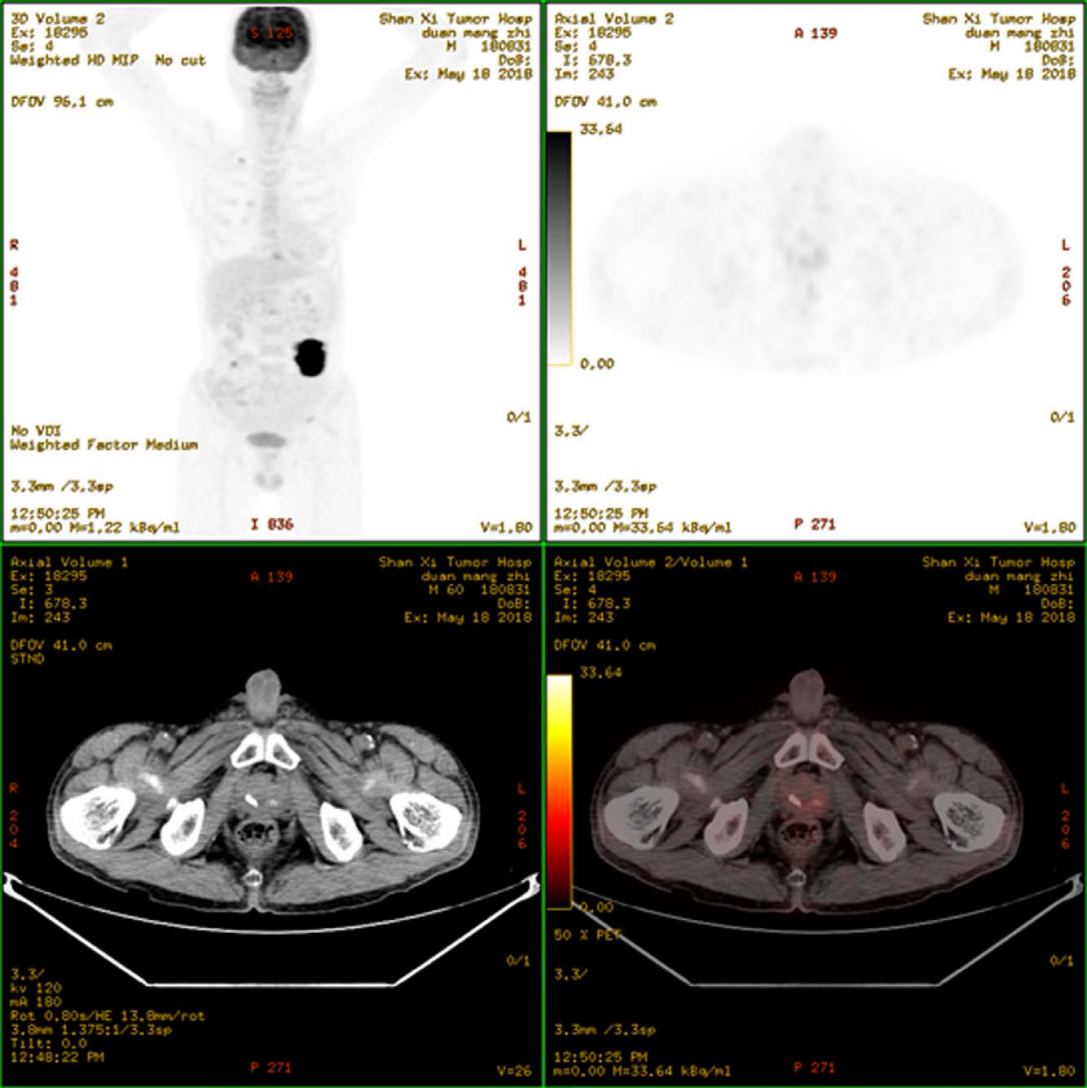
^18^F-FDG PET/CT scan before chemotherapy. ^18^F-FDG PET/CT = ^18^F-fluorodeoxyglucose positron emission tomography/computed tomography.

**Figure 2.**
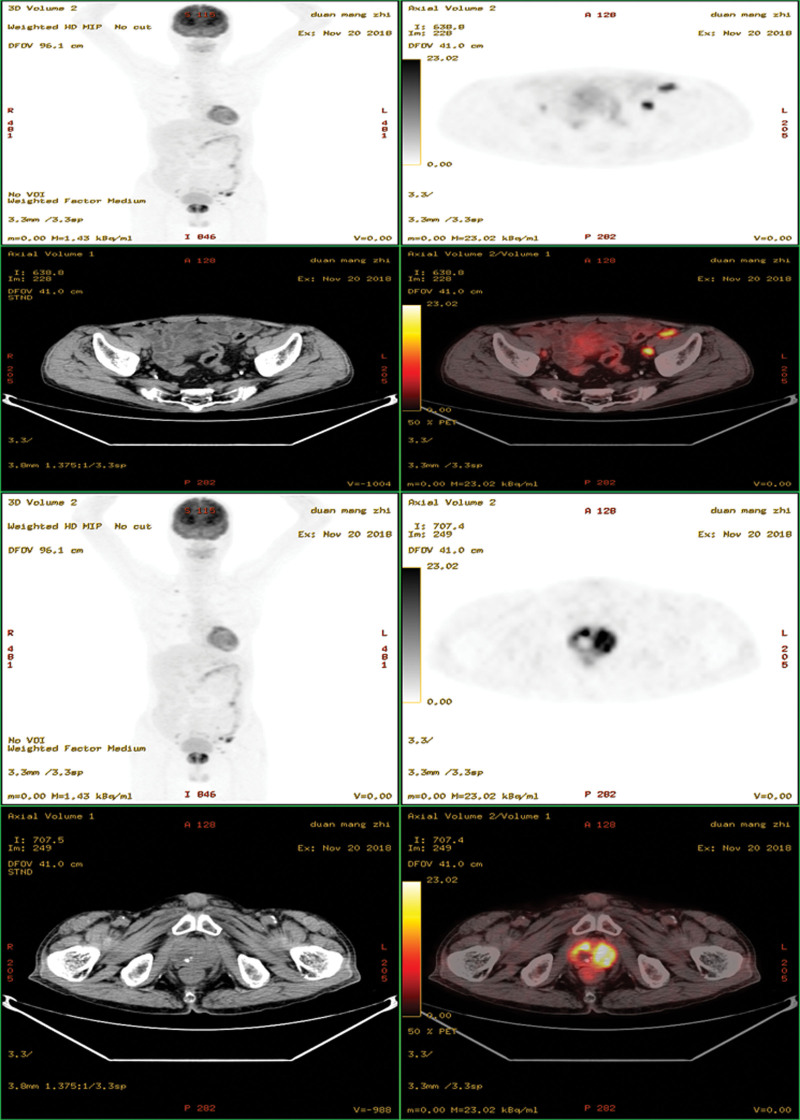
Efficacy assessment after 6 cycles of chemotherapy.

**Figure 3.**
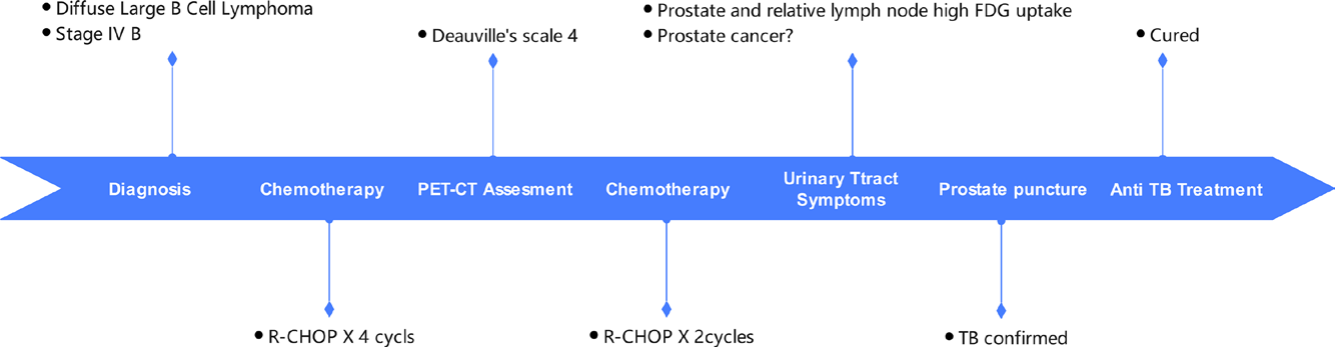
Timeline of the case. FDG = fluorodeoxyglucose, PET-CT = positron emission tomography-computed tomography, R-CHOP = An Immunochemotherapy Regimen Consisting of Rituximab and Four Chemotherapy Drugs, TB = tuberculosis.

A 60-year-old male presented to our hospital with decreased appetite and insufficient food intake of half the normal amount for four months. The patient had no fever, night sweats, abdominal pain or diarrhea, but reported weight loss of 6 kg within 6 months. Physical examination revealed a 5 × 5 cm mass in the left abdomen, and the superficial lymph nodes were not affected. The patient suffered from TB and was cured 30 years ago. Fecal occult blood was positive. Enteroscopy revealed intestinal occupation, and biopsy was performed. The pathology examination revealed submucosal diffuse distribution of medium-sized lymphoid cells, which was consistent with DLBCL. Immunohistochemical staining showed CD20+++, Ki67 about 60%+, MUM1+, Bcl-2+, c-myc+, CD3 partial+, CD56−, CD10−, CD21 scattered+, and Epstein-Barr Virus Encoded RNA−. Fluorescence in situ hybridization showed no rearrangement in *Bcl-2, Bcl-6*, and *c-myc* genes. Blood routine examination showed hemoglobin 90 g/L; lactate dehydrogenase 222 U/L, β2 microglobulin 3.17 mg/L. ^18^F-fluorodeoxyglucose (FDG) positron emission tomography/computed tomography (PET/CT) scan was performed for initial staging (Fig. 1). The jejunal wall of the left upper abdomen showed thickening and increased metabolism, with the maximum thickness of 2.3 × 6.4 cm and standardized uptake value (SUV)max was 24.01. Multiple lymph nodes were observed in bilateral renal hilum, left external iliac vessels, and left inguinal region, resulting in slightly increased metabolism. Multiple lymph node calcifications were observed in mediastinum, pulmonary hilum, abdominal cavity, retroperitoneum and bilateral iliac vessels, along with prostate calcification. Combined with pathological imaging and other examinations, non-Hodgkin’s lymphoma (diffuse large B cell type), stage IV B was confirmed. Four cycles of R-CHOP regimen (rituximab 375 mg/m^2^ d0; cyclophosphamide 750 mg/m^2^ d1; adriamycin 50 mg/m^2^ d1; vindesine 4 mg d1; prednisone 100 mg d1–5) were given to the patient. Chemotherapy was well tolerated. Interim ^18^F-FDG PET/CT scan evaluation revealed no significant thickening in the intestinal wall, and the metabolism was significantly reduced, SUVmax was 3.64. The prostate was normal in size and shape, with multiple irregular high-density calcifications. Lymph node showed increased metabolism near the external iliac vessels on the left, about 0.8 × 0.9 cm in size, SUVmax was 3.34. Deauville’s five-point scale was 4 score (Figure S1, Supplemental Digital Content, http://links.lww.com/MD/M647). After 6 cycles of chemotherapy, the patient developed acute urination, frequent urination and urinary incontinence, without fever, night sweats, or weight loss. Prostatitis was suspected, which was alleviated after oral administration of finasteride. ^18^F-FDG PET/CT examination showed that the prostate metabolic volume was increased, the maximum level was 5.1 × 4.5 cm, with increased diffuse FDG uptake, SUVmax was 18.5, which were consistent with the signs of prostate cancer. Multiple metabolically increased lymph nodes were observed in the bilateral iliac region, with a maximum of 1.7 × 1.5 cm and SUVmax was 12.79. Moreover, multiple metabolically increased lymph nodes were observed in the left inguinal region, with a maximum of 1.0 × 0.8 cm and SUVmax was 4.17. Lymph node metastasis was considered (Fig. 2). The tumor markers for prostate cancer were negative. Ultrasound-guided transrectal prostate biopsy was performed. The pathology examination revealed numerous inflammatory cell infiltrations in the prostate stroma, with epithelioid granuloma and necrosis in some areas. Special staining results indicated acid-fast staining+. Immunohistochemistry revealed CD20-, CD3 partial+, Bcl-2-, Ki67 about 10%+, which was consistent with TB (Figure S2, Supplemental Digital Content, http://links.lww.com/MD/M648). A clinical diagnosis of prostatic TB was subsequently made and the patient was cured after standard anti-TB treatment. The patient’s treatment process is depicted in Figure 3.

## 3. Detailed results

The ^18^F-FDG PET/CT scan performed for initial staging revealed a focal area of increased FDG uptake in the prostate, which was initially interpreted as indicative of a prostate lymphoma involvement. However, the ultrasound-guided prostate biopsy yielded results consistent with TB, characterized by the presence of epithelioid granuloma and necrosis, and positive acid-fast staining. The immunohistochemistry results were also suggestive of TB, with CD20-, CD3 partial+, Bcl-2-, and Ki67 about 10%+.

Following 6 cycles of chemotherapy, the patient experienced acute urination symptoms, and a repeat ^18^F-FDG PET/CT scan showed increased metabolic activity in the prostate and multiple lymph nodes, which raised concerns for prostate and lymph node involvement of lymphoma. However, the prostate cancer tumor markers were negative, and the biopsy results confirmed the diagnosis of prostatic TB rather than cancer.

The patient was subsequently treated with standard anti-TB therapy and achieved a complete cure. The timeline of the case, including the diagnosis, treatment, and follow-up, is depicted in Figure 3, illustrating the complexity of differentiating TB from malignancy in patients with hematological tumors.

## 4. Discussion

Lymphoma is a malignant tumor originating from the lymphatic hematopoietic system, usually presenting with enlarged lymph nodes as the initial symptom, but it can invade any organ. TB is one of the major causes of death due to infectious diseases worldwide, and can invade all organs of the body. Although TB has been controlled to some extent in recent years, its incidence remains relatively high. Moreover, the incidence of TB in patients with malignant tumors is high, especially those with hematological malignancy. Hypermetabolic lesions of prostate appear during the treatment of lymphoma, which makes diagnosis and treatment difficult. The incidence of prostate TB is very low, and there are no specific clinical manifestations in the early stage, so it is difficult to distinguish it from prostate malignant tumor and prostatitis, thereby leading to a high misdiagnosis rate. The report of the diagnosis and treatment of this case is aimed to remind clinicians to consider the possibility of TB when diagnosing and treating neoplastic diseases.

TB can be easily misdiagnosed as lymphoma,^[1]^ and could also co-exist with lymphoma.^[2]^ Patients with lymphoma are vulnerable to TB.^[3]^ In a population-based study of more than 450,000 patients diagnosed with cancer in Israel,^[3]^ the incidence of TB following a cancer diagnosis was higher among patients with hematological malignancies those with solid tumors. Lymphoma and MDS/MPN patients have the highest risk of developing TB. The increased susceptibility to TB among patients with hematological malignancies is possibly related to the suppression of the cellular immune system, which may lead to reactivation of residual TB bacilli in the body (endogenous), or the invasion of external TB bacilli (exogenous).

^18^F-FDG PET/CT is a noninvasive imaging method that has been widely used in the diagnosis, staging, and prognostic assessment of lymphoma and other malignant tumors as well as characterize tumors at the molecular level.^[4]^ FDG is currently the most widely used PET imaging agent, but it is not a tumor-specific agent, and its uptake by active TB could be prominent, leading to diagnostic difficulties. The reasons for the misdiagnosis of this patient were as follows: rarity of prostatic TB; elderly men often suffer from prostatitis. Both prostatic TB and prostatitis can present with lower urinary tract obstruction symptoms, and 5 α-reductase inhibitor can alleviate these symptoms to a certain extent. So it is easy to misdiagnose prostatic TB as common prostatitis; the uptake of ^18^F-FDG in prostatic TB is comparable with prostate cancer, and there is no characteristic imaging features of prostatic TB in PET/CT.

This patient was diagnosed with TB 30 years ago and was cured by surgery and anti-TB treatment. PET/CT examination suggested lymph node calcifications in mediastinum, pulmonary hilum, peritoneal cavity, retroperitoneum and bilateral iliac vessels, and the formation of prostate calcification foci, but negligible FDG uptake in the prostate. PET/CT evaluation after 4 cycles of immunochemotherapy suggested normal prostate size and morphology, along with elevated FDG uptake (SUVmax = 4.66), so inflammation was considered. PET/CT examination after 6 cycles suggested increased prostate volume and increased diffused FDG uptake (SUVmax = 18.5). The expansion of prostate TB was detected during immunochemotherapy for lymphoma, which was consistent with a previous report. Consequently, during the treatment of lymphoma, it is necessary to consider the incidence of concurrent or secondary TB, specifically in those with the medical history of TB. Moreover, the biopsy of new lesions should be considered after the original lesions have subsided. Diagnostic examinations including tuberculin test and TB antibody are needed in addition to the biopsy.

In this case, the patient was diagnosed with prostate TB by transrectal ultrasound-guided prostate biopsy, which excluded prostate cancer and the involvement of lymphoma, thus avoiding misdiagnosis and mistreatment. The diagnosis and treatment of neoplastic diseases should be carefully monitored in the differential diagnosis of TB. Transrectal prostate ultrasound-guided puncture is an important diagnostic method for the differential diagnosis of prostate tumor and prostate TB.

## 5. Limitations

Although this case report provides valuable insights into the potential for prostate TB to mimic malignancies on PET/CT scans, there are several limitations to consider. Firstly, the study is based on a single patient’s case, which limits the generalizability of the findings. Secondly, the misdiagnosis in this instance highlights the need for further research into the development of more specific imaging markers for differentiating TB from prostate cancer. Additionally, the study does not address the full spectrum of TB manifestations or the impact of immunocompromised states on the accuracy of PET/CT scans. Despite these limitations, the case serves as a critical reminder of the importance of a comprehensive clinical approach in the diagnosis and management of patients with suspected malignancies.

## Author contributions

**Writing – original draft:** Min Bai, Qinchuan Yu, Ling Yuan, Meijing Zheng.

**Writing – review & editing:** Qinchuan Yu, Meijing Zheng, Liping Su, Lieyang Wang.

**Conceptualization:** Ling Yuan.

**Resources:** Ling Yuan, Yuanyuan Zhao.

## Supplementary Material

**Figure:**
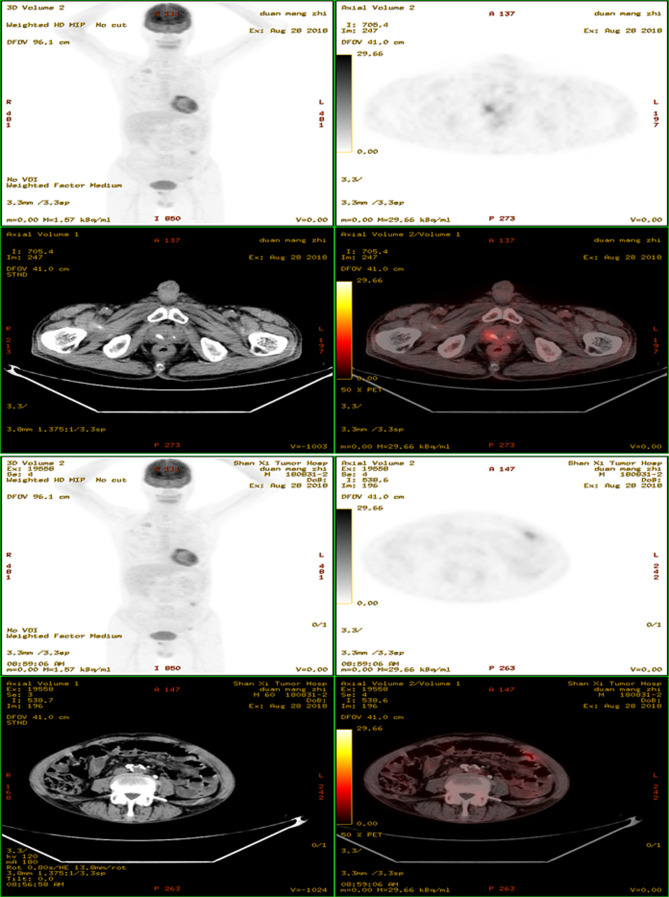


**Figure:**
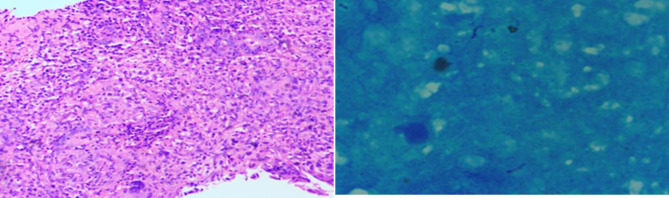


## References

[1] Akdogan RA Halil Rakici AA Gungor S Bedir R Akdogan E. F-18 fluorodeoxyglucose positron emission tomography/computed tomography findings of isolated gastric tuberculosis mimicking gastric cancer and lymphoma. Euroasian J Hepatogastroenterol. 2018;8:93–6. 29963474 10.5005/jp-journals-10018-1270 PMC6024047

[2] Jehanno N Cassou-Mounat T Vincent-Salomon A Luporsi M Bedoui M Kuhnowski F. PET/CT imaging in management of concomitant Hodgkin lymphoma and tuberculosis—a problem solver tool. Clin Case Rep. 2018;6:232–4. 29375875 10.1002/ccr3.1248 PMC5771937

[3] Ganzel C Silverman B Chemtob D Ben SA Wiener-Well Y. The risk of tuberculosis in cancer patients is greatest in lymphoma and myelodysplastic syndrome/myeloproliferative neoplasm: a large population-based cohort study. Leuk Lymphoma. 2019;60:720–5. 30188229 10.1080/10428194.2018.1499904

[4] Le Gouill S Casasnovas RO. Interim PET-driven strategy in de novo diffuse large B-cell lymphoma: do we trust the driver? Blood. 2017;129:3059–70. 28416502 10.1182/blood-2016-05-672196

